# Dual guidance structure for evaluation of patients with unclear diagnosis in centers for rare diseases (ZSE-DUO): study protocol for a controlled multi-center cohort study

**DOI:** 10.1186/s13023-022-02176-1

**Published:** 2022-02-14

**Authors:** Helge Hebestreit, Cornelia Zeidler, Christopher Schippers, Martina de Zwaan, Jürgen Deckert, Peter Heuschmann, Christian Krauth, Monika Bullinger, Alexandra Berger, Mark Berneburg, Lilly Brandstetter, Anna Deibele, Jan Dieris-Hirche, Holm Graessner, Harald Gündel, Stephan Herpertz, Gereon Heuft, Anne-Marie Lapstich, Thomas Lücke, Tim Maisch, Christine Mundlos, Andrea Petermann-Meyer, Susanne Müller, Stephan Ott, Lisa Pfister, Julia Quitmann, Marcel Romanos, Frank Rutsch, Kristina Schaubert, Katharina Schubert, Jörg B. Schulz, Susann Schweiger, Oliver Tüscher, Kathrin Ungethüm, Thomas O. F. Wagner, Kirsten Haas, ZSE-DUO working group, Federica Akkaya, Christine Babka, Lavinia Bârlescu, Anja Bärsch-Michelmann, Astrid Bergbreiter, Janika Blömeke, Leonie Böhm, Benita Böttger, Birgit Braun, Folke Brinkmann, Vanessa Britz, Holger Cario, Melisa Celiker, Moritz de Greck, Klaus-Michael Debatin, Katrin Dillmann-Jehn, Max Ertl, Monika Ettinger, Jutta Eymann, Jörg Frommer, Martina Gabrian, Anja Glode, Vega Gödecke, Corinna Grasemann, Eva Grauer, Helmut Greger, Astrid Haas, Martina Haase, Lea Haisch, Isabel Heinrich, Melissa Held, Julia Hennermann, Anne Herrmann-Werner, Julian Hett, Bettina Hilbig, Laura Holthöfer, Christiane Imhof, Titus Jacob, Florian Junne, Stefanie Karl, Jan Kassubek, Lisa Kick, Kevin-Thomas Koschitzki, Heike Krassort, Christian Kratz, Kaja Kristensen, Birgit Kropff, Julia Kuhn, Philipp Latzko, Thomas Loew, Delia Lorenz, Albert C. Ludolph, Isabell Meyer dos Santos, Torsten Meyer, Klaus Mohnike, Martina Monninger, Thomas Musacchio, Amalia Nicole Nanciu, Margret Nießen, Mariell Nöhre, Aikaterini Papagianni, Christina Pfeifer-Duck, Lea-Sophie Piduhn, Carina Rampp, Antonia Richter, Olaf Rieß, Annika Schmidt, Simone Schneider, Ludger Schoels, Martina Schwalba, Udo Selig, Astrid Spangenberger, Alexandra Sroka, Toni Steinbüchel, Sebastian Stösser, Steffi Suchant, Matthias Vogel, Daniela Volk, Christoph Vollmuth, Solange Volnov, Sabrina Walter, Bodo Warrings, Christine Weiler, Stefanie Witt, Kamil Kajetan Zajt, Lena Zeltner, Karola Zenker, Kailun David Zhang, Stephan Zipfel

**Affiliations:** 1 Center for Rare Diseases - Reference Center Northern Bavaria (ZESE), University Hospital Würzburg, Josef-Schneider-Str. 2, 97080 Würzburg, Germany; 2 Center for Rare Diseases, Hannover Medical School, Carl-Neuberg-Strasse 1, 30625 Hannover, Germany; 3 Center for Rare Diseases, RWTH Aachen University Hospital, Pauwelsstraße 30, 52074 Aachen, Germany; 4 Department of Psychosomatic Medicine and Psychotherapy, Hannover Medical School, Carl-Neuberg-Strasse 1, 30625 Hannover, Germany; 5 Department of Psychiatry, Psychosomatics and Psychotherapy, Center of Mental Health, University Hospital, Margarete-Höppel-Platz 1, 97080 Würzburg, Germany; 6 Institute of Clinical Epidemiology and Biometry, University of Würzburg, Josef-Schneider-Str. 2, 97080 Würzburg, Germany; 7 Center for Health Economics Research Hannover and Institute for Epidemiology, Social Medicine and Health Systems Research, Hannover Medical School, Carl-Neuberg-Strasse 1, 30625 Hannover, Germany; 8 Department for Medical Psychology, University Medical Center Hamburg-Eppendorf, Martinistr. 52, 20246 Hamburg, Germany; 9 Frankfurt Reference Centre for Rare Diseases, University Hospital Frankfurt, Goethe University Frankfurt, Theodor-Stern-Kai 7, 60590 Frankfurt am Main, Germany; 10 Center for Rare Diseases Regensburg, University Hospital Regensburg, Franz-Josef-Strauss-Allee 11, 93053 Regensburg, Germany; 11 Department of Psychosomatic Medicine and Psychotherapy, University Hospital Magdeburg, Leipziger Straße 44, 39120 Magdeburg, Germany; 12 Department of Psychosomatic Medicine and Psychotherapy, LWL-University Clinic, Ruhr-University Bochum, Alexandrinenstraße 1-3, 44791 Bochum, Germany; 13 Centre for Rare Disease, University Hospital Tübingen, Calwerstr. 7, 72076 Tübingen, Germany; 14 Department of Psychosomatic Medicine and Psychotherapy, University Hospital Ulm, Albert.-Einstein-Allee 23, 89081 Ulm, Germany; 15 Department of Psychosomatic Medicine Und Psychotherapy, University Hospital Münster, Albert-Schweitzer-Campus 1, 48149 Münster, Germany; 16 University Hospital of Pediatrics and Adolescent Medicine, Ruhr-University Bochum, Alexandrinenstr. 5, 44791 Bochum, Germany; 17 Allianz Chronischer Seltener Erkrankungen (ACHSE) e.V., c/o DRK Kliniken Berlin Mitte, Dronheimer Str. 39, 13359 Berlin, Germany; 18 Department of Hematology, Oncology, Hemostaseology and Stem Cell Transplantation, Section Psychooncology, Center for Integrated Oncology – Aachen, Medical Faculty, RWTH Aachen University, Aachen, Germany; 19 Department of Neurology, University of Ulm, Oberer Eselsberg 45, 89081 Ulm, Germany; 20 Department of Psychosomatics and Psychotherapy, University Hospital Tübingen, Osianderstraße 5, 72076 Tübingen, Germany; 21 Department of Child Psychiatry, Psychosomatics and Psychotherapy, Center of Mental Health, University Hospital Würzburg, Margarete-Höppel-Platz 1, 97080 Würzburg, Germany; 22 Department of General Pediatrics, Muenster University Children’s Hospital and Center for Rare Diseases, Muenster University Hospital, Albert-Schweitzer-Campus 1, Gebäude A1, 48149 Münster, Germany; 23 Central German Competence Network for Rare Diseases, University Hospital Magdeburg, Leipziger Straße 44, 39120 Magdeburg, Germany; 24 Department of Neurology, RWTH Aachen University, Pauwelsstraße 30, 52074 Aachen, Germany; 25 Centre for Rare Disease and Institute for Human Genetics, University Medical Center, Johannes Gutenberg University, 55131 Mainz, Germany; 26 Centre for Rare Disease and Department of Psychiatry and Psychotherapy, University Medical Center, Johannes Gutenberg University, 55131 Mainz, Germany; 27 Universitätsklinikum Ulm, Ulm, Germany; 28 Medizinische Hochschule Hannover, Hanover, Germany; 29 IKK gesund plus, Magdeburg, Germany; 30 Universitätsklinikum Regensburg, Regensburg, Germany; 31 Universitätsklinikum Frankfurt, Frankfurt, Germany; 32 UKE Hamburg, Hamburg, Germany; 33 Uniklinik RWTH Aachen, Aachen, Germany; 34 Universitätsklinikum Magdeburg, Magdeburg, Germany; 35 Universität Würzburg, Würzburg, Germany; 36 Universitätskinderklinik Bochumm, Bochum, Germany; 37 Universitätsklinikum Würzburg, Würzburg, Germany; 38 LWL-Universitätsklinikum Bochum, Bochum, Germany; 39 Universitätsklinikum Tübingen, Tübingen, Germany; 40 AOK Hessen, Offenbach am Main, Germany; 41 Universitätsklinikum Münster - UKM, Münster, Germany; 42 Universitätsmedizin Der Johannes Gutenberg-Universität Mainz, Mainz, Germany; 43 Techniker Krankenkasse, Hamburg, Germany; 44 ACHSE e.V., Berlin, Germany

**Keywords:** Rare diseases, Undetermined symptoms, Unclear diagnosis, Mental health disorders, Cohort study

## Abstract

**Background:**

In individuals suffering from a rare disease the diagnostic process and the confirmation of a final diagnosis often extends over many years. Factors contributing to delayed diagnosis include health care professionals' limited knowledge of rare diseases and frequent (co-)occurrence of mental disorders that may complicate and delay the diagnostic process. The ZSE-DUO study aims to assess the benefits of a combination of a physician focusing on somatic aspects with a mental health expert working side by side as a tandem in the diagnostic process.

**Study design:**

This multi-center, prospective controlled study has a two-phase cohort design.

**Methods:**

Two cohorts of 682 patients each are sequentially recruited from 11 university-based German Centers for Rare Diseases (CRD): the standard care cohort (control, somatic expertise only) and the innovative care cohort (experimental, combined somatic and mental health expertise). Individuals aged 12 years and older presenting with symptoms and signs which are not explained by current diagnoses will be included. Data will be collected prior to the first visit to the CRD’s outpatient clinic (T0), at the first visit (T1) and 12 months thereafter (T2).

**Outcomes:**

Primary outcome is the percentage of patients with one or more confirmed diagnoses covering the symptomatic spectrum presented. Sample size is calculated to detect a 10 percent increase from 30% in standard care to 40% in the innovative dual expert cohort. Secondary outcomes are (a) time to diagnosis/diagnoses explaining the symptomatology; (b) proportion of patients successfully referred from CRD to standard care; (c) costs of diagnosis including incremental cost effectiveness ratios; (d) predictive value of screening instruments administered at T0 to identify patients with mental disorders; (e) patients’ quality of life and evaluation of care; and f) physicians’ satisfaction with the innovative care approach.

**Conclusions:**

This is the first multi-center study to investigate the effects of a mental health specialist working in tandem with a somatic expert physician in CRDs. If this innovative approach proves successful, it will be made available on a larger scale nationally and promoted internationally. In the best case, ZSE-DUO can significantly shorten the time to diagnosis for a suspected rare disease.

*Trial registration* ClinicalTrials.gov; Identifier: NCT03563677; First posted: June 20, 2018, https://clinicaltrials.gov/ct2/show/NCT03563677.

## Introduction

In Europe, a disease is classified as rare if less than 5 in 10.000 citizens are affected. It is estimated that about 27–36 million people in the member states of the European Union [1] and about 4 million people in Germany [2] suffer from a rare disease. With more than 7.000 distinct rare diseases described so far [3] and most of these affecting only a few people in Europe, establishing a diagnosis is often difficult. In many cases, even with finally established rare diseases, it takes years to name the health condition and to initiate targeted treatment [4, 5].

Rare diseases often affect multiple organ systems and vary in their manifestations among individuals. In several disorders such as 22q11 deletion syndrome, psychological symptoms are part of the clinical manifestations of the disease itself [6]. However, given the progressive and debilitating course of many rare diseases and the typically long and frustrating way to diagnosis, individuals with rare diseases may also develop a co-morbid mental disorder [5]. Furthermore, patients with a mental or behavioral disorder—possibly associated with a common health condition—may be suspected to suffer from a rare disease. Irrespective of the prevalence of the underlying disease—be it rare or not –, a (co-)morbidity with a mental disorder may lead to a more complex symptomatology thereby further delaying the diagnosis and adequate treatment.

In 2013, a National Plan of Action for People with a Rare Disease was presented in Germany which—among other measures—called for structures and processes to improve the diagnostic process in people with a suspected rare disease but yet undiagnosed health condition [7]. Subsequently, most German Centers for Rare Diseases (CRDs) established outpatient clinics for undiagnosed patients. In these clinics, patients are seen by a specialist from a somatic discipline such as internal medicine, neurology, pediatrics etc. If psychiatric and/or psychosomatic expertise is required, the patient is usually referred to respective specialists. Although in the authors’ experience the majority of patients presenting to a clinic for undiagnosed cases are severely distressed and present with mental health problems, only a few are eventually seen by a respective specialist and even fewer come back for evaluation of a potential rare disease. In fact, patients frequently report the impression of not being taken seriously, feel relegated and are highly suspicious or even refuse to be evaluated by a mental health specialist. Therefore, a close collaboration of both somatic and mental health specialists during the diagnostic process and subsequent treatment decisions might significantly improve patient care. Thus, the objective of the ZSE-DUO project is to assess the benefits of a mental health specialist working in tandem with an expert in somatic medicine at a CRD.

## Methods

### Study design

**Fig. 1:**
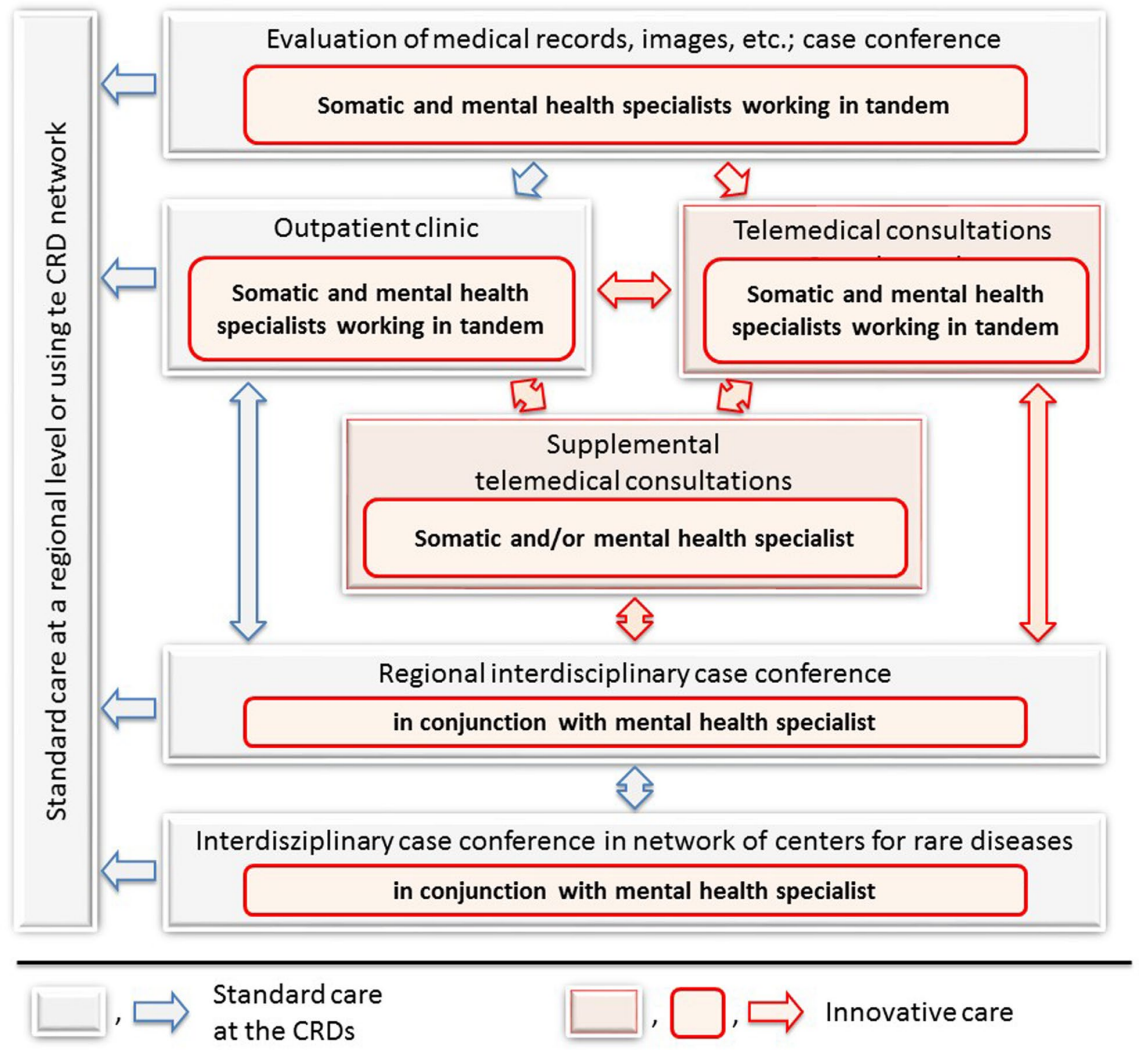
Standard diagnostic approach employed for people with a suspected rare disease in Centers for Rare Diseases and additional innovative elements established in the ZSE-DUO project

ZSE-DUO is a multi-center, prospective, controlled trial with a two-phase cohort design (Clinicaltrials.govIdentifier: NCT03563677). Eleven CRDs in Germany recruit individuals with a suspected rare disease but unclear diagnosis. Study participants of the control group are consecutively enrolled during the first 12 months of the project and are diagnosed and treated according to the Standard Care (SC) procedures. Participants of the intervention group recruited during the following 16 months receive the Innovative Care (IC) procedures which integrate the dual expert components (see Fig. 1). For recruitment of the IC group, the period had to be extended from 12 months—as originally planned—to 16 months because of stops in recruitment during the COVID-19 pandemic. Due to the nature of the patients, settings and interventions a sequential cohort design with the IC group following the SC group was preferred over a randomized trial, as blinding of participants and team members is not possible and cross-contamination between groups might have occurred in a randomized design.

In addition, to understand possible selection bias, outcomes of patients seen in outpatient clinics of participating CRDs 9 months prior to the start of the project, e.g. (confirmed) diagnoses and time to diagnosis, are retrospectively assessed and compared to the outcome of the SC group in these centers. By this means, unintended changes in standard care with the start of the ZSE-DUO project can be detected.

### Study population

Participants are recruited by 11 CRDs associated with university hospitals in the cities of Aachen, Bochum, Frankfurt, Hannover, Magdeburg, Mainz, Münster, Regensburg, Tübingen, Ulm and Würzburg. Recruitment is supported by a collaboration with the National Alliance of Chronic Rare Diseases Germany (ACHSE e.V.) representing many rare disease organizations.

Individuals with a suspected rare disease but unclear diagnosis who approach one of the participating centers or are referred to one of these centers by their treating physician are assessed for eligibility to participate in the study.

Inclusion criteria for participation in the project are:

i) first contact with a participating CRD,
ii) suspicion of a rare disease but no established diagnosis,
iii) attending the CRD as an outpatient, and
iv) written informed consent.

Patients are excluded from the study if one or more of the following exclusion criteria apply:

i) age < 12 years,
ii) incomplete medical records available to the CRD at the time of presentation (records must include medical summary letters, imaging studies, blood tests etc.), and
iii) pre-diagnosed disease(s) explaining the symptomatic spectrum presented.

Furthermore, due to the funding of the project, patients with a private health insurance (ca. 10.5% of German patients) cannot be enrolled.

### Randomization

No randomization will be conducted since allocation to control and intervention conditions will occur based on timing of recruitment. However, participants will not be informed about allocation when informed consent is obtained. Thus, participants consent to all innovative care components even if they will receive standard care only.

### Control (SC) and intervention (IC) group

Invitation for a clinic visit at a CRD follows the established procedures in each CRD which are based on the national plan of action for people with rare diseases in Germany [7]. After collecting a complete information package including medical records, imaging studies, a physician referral and structured information from the patient, all information is evaluated by an interdisciplinary team to discuss symptomatology and potentially underlying diagnoses as well as eligibility to participate in the trial. Thereafter, patients are invited for a visit to one of the 11 participating CRD outpatient clinics for undiagnosed patients. All patients invited for a clinic visit and suitable for the trial according to the inclusion and exclusion criteria are asked to participate in the study.

#### Standard care (control group)

At the outpatient clinic, the medical history is taken and the patient is examined by a physician from a “somatic” discipline such as a specialist in internal medicine, neurology, or pediatrics. Then, additional diagnostic evaluations such as blood testing, imaging, or a consultation of another expert are performed if needed to establish a diagnosis. Interdisciplinary case discussions at a local level are used to include more expertise from additional medical disciplines including mental health specialists for selected patients in whom the diagnosis remains unclear. The referring physician and the patient receive a medical letter summarizing all information and providing recommendations for further evaluations and/or therapy.

#### Innovative care (intervention group)

The innovative care includes all aspects of standard care, yet involves dual expertise both from somatic and mental health experts working in tandem. All medical decisions from the diagnostic approach to care procedures at the CRD involve both disciplines (see Fig. 1). This approach is applied to the entire care process: the evaluation of patient records before the patient is seen, the outpatient visit to the CRD, the care following the visit as well as the writing of the medical letter. Case conferences at a local and national level using videoconferencing allow including additional expertise in the medical evaluations.

At the initial clinic visit, the patient is introduced to the innovative care approach, preferably by both somatic and mental health physicians. It is made clear that the patient will meet both physicians during the visit, that they will work in tandem and will both obtain medical and family histories. The complete somatic medical examination is supplemented by diagnostic tests and procedures targeted to narrow down or confirm suspected somatic diagnoses. The mental health specialist will add the psychosocial history and a psychiatric/psychosomatic evaluation including a standardized diagnostic interview for mental disorders (Mini-DIPS Open Access Interview) [8, 9] and a screening questionnaire for personality disorders (PSS-K) [9, 10]. The Mini-DIPS Open Access allows identifying the most important mental disorders based on the criteria of DSM-IV and ICD-10 with the help of the diagnostic interview for mental disorders. Reliability and validity have been tested before. Following the recommendations, a two-stepped procedure was employed. At first, participants were asked a screening question regarding the specific mental disorder. If the question was answered positively, further diagnostic questions followed to confirm the diagnosis. All mental health experts received standardized training in applying the Mini-DIPS. The goal of this evaluation is to clarify if (some) symptoms of the patients can be explained by mental disorders or severe psychological distress (e.g. sleep disorders in depression, tachycardia during anxiety attacks). During case conferences symptoms are explored avoiding dichotomization of unexplained somatic complaints into somatic and mental categories. In other words, based on a multifactorial concept of symptom genesis, symptoms are collected without a priori defined attribution of particular symptoms to either a somatic or a psychological genesis.

Furthermore, both physicians have the option to use telemedicine including videoconferencing to communicate with the patient before and after clinic visit(s) (e.g. hints for severe disorder or suicidal tendency; need for urgent medical/psychiatric treatment; planning or follow-up of clinic visit). To facilitate the transition to standard care for mental health conditions and bridge the—often quite long—waiting period for specialist care, the mental health specialist is encouraged to offer appointments via a videoconferencing tool for patients with a mental (co-)morbidity.

### Recruitment and study procedures

**Fig. 2:**
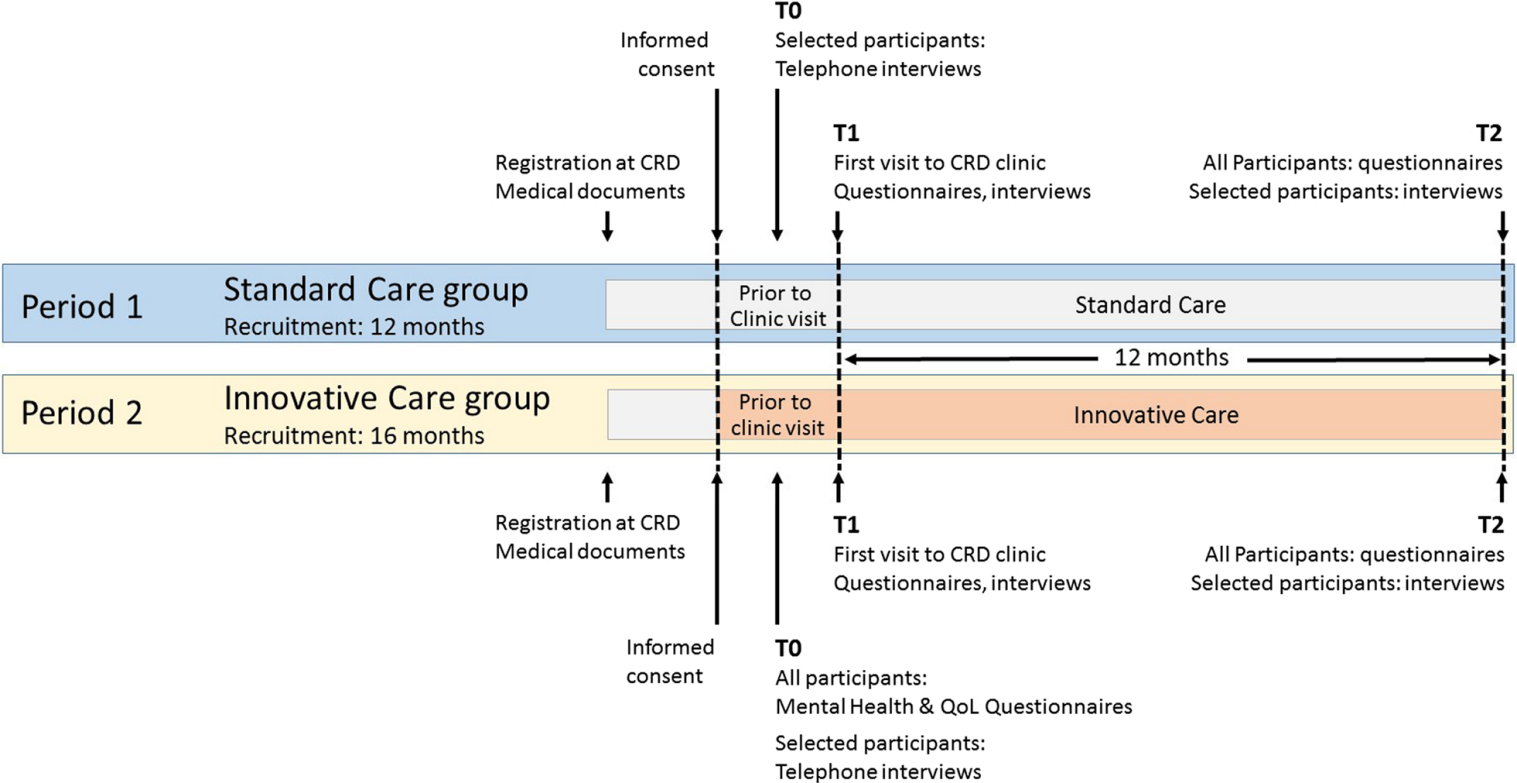
Timeline of assessments at timepoints T0, T1 and T2 during the study. Recruitment and delivery of care in the standard care and the innovative care groups occurred in consequtive time periods

Figure 2 provides an overview of assessment time points during the ongoing study. This project is currently ongoing. The first patient was enrolled on October 12, 2018 and the estimated study completion date will be January 2022. In the first phase, the patients were consecutively enrolled into the SC group only. In the second phase, additional patients were consecutively enrolled into the IC group. Prior to the initial clinic visit (T0), patients complete a set of questionnaires and 10% of the patient enrolled are contacted by phone to assess their expectations regarding diagnosis, treatment, and care. Shortly after the clinic visit, the symptoms of the patients are recorded using human phenotype ontology coding (HPO). The HPO is a globally standardized vocabulary for phenotypic abnormalities that occur in human diseases [11]. Newly made diagnoses are also documented. Follow-up assessments are conducted 12 months after the initial clinic visit.

Table 1 provides an overview over patient-related data collected during the project and their mode of assessment.

**Table 1:** Patient data collected and instruments used at baseline and 12-month follow-up

	T0 Prior to first clinic visit	T1 At first clinic visit	T2 At 12 month follow-up
Socio-demographic data	CRD patient questionnaire	Socio-demographic history (family status, education)	–
Employment status (includes unemployment and disability)	CRD patient questionnaire	Social history (employment status, salaries), ZSE-DUO health economics questionnaire (changes in employment status, salaries)	ZSE-DUO health economics questionnaire (changes in employment status, salaries)
Signs and symptoms	CRD patient questionnaire, medical summary from referring physician, medical letters	Medical history, physical examination, psychopathological status^a^, diagnostic procedures	–
Prior diagnoses	medical summary by referring physician, medical letters	Medical history	–
New diagnoses / symptomatology explained	–	Medical history, physical examination, mental disorders^a^, diagnostic procedures	Medical information available after clinic visit
Successful transition to standard care delivered by other health care providers	–	–	Medical information available after clinic visit
Quality of life (QoL)	EQ-5D-5L, SF-12 (adults), Kidscreen-10 (youth) ^a^, qualitative telephone interviews	EQ-5D-5L, SF-12 (adults), life satisfaction, Kidscreen-10 (youth)	EQ-5D-5L, SF-12 (adults), life satisfaction, Kidscreen-10 (youth), qualitative telephone interviews
Mental status	PHQ-15, GAD-7, DSS-4, SCL-K-9, SDQ (youth) ^a^	PHQ-15, GAD-7, DSS-4, SCL-K-9, SDQ (youth), MINI-DIPS^a^, PSS-K^a^	PHQ-9, GAD-7, SDQ (youth)
Health economics data	–	ZSE-DUO health economic questionnaire (adaption of FIMA-questionnaire)	ZSE-DUO health economic questionnaire (adaption of FIMA-questionnaire)
Expectations^b^	qualitative telephone interviews^b^	–	–
Satisfaction	–	–	ZUF-8, qualitative telephone interviews^b^
Health insurance data	–	Selected items	Selected items

Abbreviations of instruments used: EQ-5D-5L, 5 dimensions 5 level quality of life (QoL) questionnaire of the EuroQol group; SF-12, Short Form Health questionnaire [12, 13]; PHQ-9, PHQ-15, Patient Health Questionnaire-9 and -15 [14–16]; GAD-7, General Anxiety Disorder-7 questionnaire [17, 18]; DSS-4, Dissociation Tension Scale [19]; SCL-K-9, Symptom Checklist [20]; SDQ, Strengths and Difficulties Questionnaire [21]; Mini-DIPS, standardized diagnostic interview for mental disorders [8, 9]; PSS-K, screening measure for the assessment of personality disorders [10]; FIMA, questionnaire for health-related resource use in the elderly population [22]

^a^in innovative care group only

^b^10% of patients only

At the end of the innovative care period, satisfaction of physicians involved with the new care will be assessed using a questionnaire specifically developed for this project. The items assessed and specific questions asked will be derived from the input of three focus groups of 8–10 CRD physicians.

### Study endpoints and measurements

Table 2 summarizes the primary and secondary endpoints of the study.

**Table 2:** Study endpoints

Primary endpoint	Proportion of patients with one or more confirmed diagnoses covering the symptomatic spectrum presented
Secondary endpoints	a) Time to diagnosis/diagnoses explaining the symptomatology of the patient
	b) Proportion of patients successfully referred from CRD to standard care
	c) Costs of diagnosis including incremental cost effectiveness ratios
	d) Identification of patients suffering from mental disorders by screening questionnaires
	e) Patients’ quality of life and evaluation of care (i.e., satisfaction with the process of diagnosis and treatment)
	f) Physicians’ satisfaction with the innovative care

The primary endpoint in ZSE-DUO is the percentage of patients for whom one or more diagnoses can be confirmed during the evaluation process that explain the symptomatic spectrum presented by the patient. The evaluation period encompasses the period between the initial clinic visit to the CRD (T1) and the 12-month follow-up (T2). The primary endpoint is assessed by using data on symptomatology and diagnosis entered by the treating physician(s) of the CRDs in a project database using electronic case report forms (eCRF).

Secondary endpoints of the project are:

- Time to diagnosis/diagnoses explaining the symptomatology of the patient. We hypothesize that the period between the initial visit to the CRD and the time of diagnosis averages 6 months with standard care, while the innovative care can shorten this period to 4.5 months. The difference of 1.5 months is considered clinically meaningful by the heads of the participating CRDs. The respective analyses will be based on data collected from the CRDs and the patients at initial clinic visit and follow-up and entered in the eCRFs. The date of diagnosis fully explaining a patient´s symptomatology will be defined by the treating physician(s) at the CRD.
- Proportion of patients successfully referred from CRD to standard care. The innovative care probably facilitates this referral resulting in more patients being specifically cared for by other health care providers within 12 months after the first visit to the CRD. A successful transition will be defined by the treating physician(s) based on patient’s responses to the structured 12-month follow-up questionnaire and information available at the respective CRD. The criterion for a successful transition is defined as at least one outpatient or inpatient visit to a physician specialized in a discipline which was recommended by the CRD and is part of available standard care.
- Costs of diagnosis and incremental cost effectiveness ratios. It is hypothesized that the innovative care will reduce the costs of the diagnostic process and positively impact incremental cost effectiveness ratios compared to standard care. Health economic analyses will address costs of the diagnostic process, QoL, incremental cost-effectiveness ratios and costs of therapy linked to confirmed diagnosis of a rare disease. From the incremental cost-effectiveness ratios, additional costs (or savings) linked to the innovative care compared with standard care may be estimated by calculating the total benefits and costs of both care models.
  To assess the costs of diagnosis and therapy, case-related processes are identified and analyzed. Based on this process analysis, used resources are identified and their economic value determined. The analysis of processes and the resource utilizations is based on surveying staff members. The financial assessment is based on established valuation rates [23, 24]. Further information on costs of diagnosis is gained from the documentation of the CRDs (e.g., individual process steps documented by the medical specialists) and patients´ surveys at baseline and 12-months follow-up (e.g., QoL). Costs of therapy (e.g., medication, contacts to the medical system) is derived from documentation of the CRDs and account data from the participating health insurances. For the latter data, secondary analyses of health insurance data from the Techniker Krankenkasse, IKK gesund plus and AOK Hessen is performed. These data encompass costs of outpatient and inpatient medical care, drugs, therapeutic remedies and aids, home-care services and medical rehabilitation as well as periods of unemployment or disability to work.
- Identification of patients suffering from mental disorders or severe distress needing to see a mental health specialist at the clinic visit by screening questionnaires, regardless of the presence or absence of a potential rare disease. Prior to the first clinical visit to the CRD, patients of the innovative care group complete a set of questionnaires (Table 1). The predictive value of these screening instruments with respect to mental disorders needing appropriate evaluation and possibly treatment will be assessed. In confirmatory factor analyses, it will be determined whether the number of questions (40 questions from 6 instruments) may be reduced to develop a suitable short questionnaire with sufficient sensitivity in the prediction of a mental disorder or severe distress. This instrument could be useful for future targeted allocation of resources for evaluations by a mental health professional in CRDs.
- Patients’ quality of life and evaluation of care. It is hypothesized that the innovative care will improve patient QoL and satisfaction with care. The hypothesis is assessed in the total sample using an established German questionnaire to measure patient satisfaction (Fragebogen zur Messung der Patientenzufriedenheit, ZUF-8) [25] and the QoL questionnaires EQ-5D from the EuroQoL-Group in all patients. Furthermore, the Short Form 12 (SF-12) [26] is administered in patients 16 years and older while the QoL-questionnaire KIDSCREEN-10 is used in patients younger than 16 years.
  In a randomly selected subsample of 68 patients per group, stratified by age (12-18 years, 19-40 years, >40 years) and sex, structured telephone interviews are conducted before the initial visit to the CRD and 12 months after the visit. The qualitative assessment will address next to the assessment of the patients perceived health (using the SF-8) [27], distress (using the NCCN Distress Thermometer (DT)) [28] and patient reported experiences and satisfaction with care (Lübecker Fragebogen-Doppelkarte) [29] the perceived effects of care at the CRD, the estimated quality of care, as well as the satisfaction with and acceptance of structure and processes of CRD care. The interviews last up to 30 minutes and will be conducted by trained staff not involved in any other activities related to care or study conduct following a manual. All interviews are recorded and transcribed for further analysis [30].
- Physicians’ satisfaction with the innovative care. It is assumed that the physicians at the CRDs involved evaluate the innovative care positively. This outcome is assessed using a newly developed questionnaire administered at the end of the innovative care period to all physicians involved in the care of the patients. For the development of the questionnaire, three focus groups were conducted, each with 4 to 8 physicians. The questionnaire addresses perceived effects of the innovative approach, factors influencing success or failure, and satisfaction with and acceptance of the innovative care.

### Sample size calculation

The primary outcome of the study is the change in the proportion of patients with one or more (confirmed) diagnoses covering the symptomatic spectrum. For the sample size calculation we assumed that the innovative care will increase the percentage of patients receiving one or more confirmed diagnosis during the evaluation process from 30% with standard care to 40%. When planning the ZSE-DUO project, no applicable published information was available on the success rate of standard care in German CRDs. Thus, the assumed 30%-rate of patients with confirmed diagnoses using standard care was based on the experience and a consensus among the leading physicians of the eleven CRDs participating in this project. An absolute increase in patients with confirmed diagnosis of 10% by innovative care was considered realistic and clinically relevant by the group so that 40% overall success rate were assumed for this care model.

Power calculations were done using Monte-Carlo simulation. Data sets were generated assuming randomly varying center specific baseline prevalence rates (with an average of 95% of rates falling between 20 and 40%) and odds-ratios (with an average of 10% of centers not experiencing any positive intervention effect). For each simulated data set, center-specific odds ratios were calculated and then summarized using random-effects meta-analysis..

Based on enrollment varying between 24 and 93 patients per center and period and assuming a drop-out rate of 20%, the inclusion of 682 participants per group resulted in an estimated statistical power of 80.8% within 100,000 simulations, with a mean prevalence across simulations of 30.3% in the SC group and of 40.3% in the IC group. Statistical power was estimated as the percentage of simulations in which the random effects meta-analytic summary estimate of center-specific odds ratios was statistically significant at the 5% level.

### Data processing and statistical analysis

Data collection and analysis are coordinated and performed by the Institute for Clinical Epidemiology and Biometry at the University of Würzburg, the Institute for Epidemiology, Social Medicine and Health Systems Research at Hannover Medical School, and the Department of Medical Psychology in Hamburg.

For the primary outcome “Proportion of patients with one or more (confirmed) diagnoses covering the full symptomatic spectrum”, a mixed logistic regression model including a fixed period effect along with random center effects and random period effects nested within centers will be employed. In a second step, models will be extended by adding personal characteristics of patients (e.g., sex and age) and interaction terms between these characteristics and period. Significant interactions would suggest modification of intervention effects by the respective characteristics. While statistical significance of main effects will be defined at the 5%-level, it will be defined at the 10% level for interactions.

Secondary analyses are carried out in an exploratory way and results will be reported with 95%-confidence intervals. According to the distribution of the variables, differences between groups are tested using the χ2 test, Fishers’ exact tests, Student's t test, Mann–Whitney *U* test as well as univariable and multivariable linear regression models and mixed regression models. The incremental cost effectiveness ratio as a measure of efficacy is calculated by dividing the additional costs by the additional outcomes (QALYs) of IC versus SC. The non-parametric approach of bootstrapping is applied to estimate 95% confidence intervals of the incremental cost effectiveness ratio. The predictive value of applied standardized screening instruments will be analyzed for identifying patients affected by mental disorders. First, via exploratory factor analysis, it will be tested if the number of items of the applied screening instruments can be reduced. The new set of items will then be tested in a confirmatory factor analysis to examine how well this model fits the current data. Based on a reduced screening instrument a new score for mental disorders will be estimated using multivariable logistic models and the predictive value will be analyzed applying Receiver Operating Characteristic Graph (ROC-Graph). Qualitative interviews will be transcribed and data will be analyzed using MAXQDA.

All statistical analyses will be performed using SPSS, STATA and SAS, respectively.

### Dissemination plan

The main results will be published in a final report according to the German Innovations Funds directive. Furthermore, the scientific results will be published in peer-reviewed scientific journals and via presentations at national and international scientific conferences. The ZSE-DUO manual detailing the structure and procedures as well as experiences developed for the somatic and mental health specialists working in tandem will be published separately.

## Discussion

This is the first multi-center study investigating the effects of a dual guidance structure involving a somatic and a mental health expert working in tandem to establish one or more diagnoses in people with a suspected rare disease. Including mental health expertise in multidisciplinary teams, i.e. caring for cancer patients, has become more and more common over the last decades. Likewise, medical programs for individuals with stroke or heart attacks and evaluation before certain procedures such as bariatric surgery or transplantation benefit from the inclusion of a mental health expert [31, 32]. Additionally, in some rare diseases such as Huntington's Disease, the assessment and treatment of cognitive, emotional and behavioral symptoms have become the standard of care and an integrative part of international treatment guidelines [33].

### Strengths

This is the first study evaluating a tandem care in CRDs. The sample size is large and we will acquire a large set of data. Due to the multicenter approach (11 centers), the study concept with the developed SOPs will be tested in centers differing in structure and procedures. Findings on possible barriers and potential for improvement can be taken into account in the final recommendations and respective manual. Furthermore, the study considers the perspectives of multiple stakeholders in the care process on satisfaction, burden, acceptability, costs, and feasibility, so that it is easier to transfer and implement the concept of two expert physicians, a somatic and a mental health specialist working in tandem, in different RDC clinics.

### Limitations

There are several limitations in study design and procedures.

First, ZSE-DUO is not a randomized controlled trial with a potential bias in selection of participants. However, randomization of participants to one of the two models of care would have posed a large risk that the standard care (control) group might have received facilitated access to the mental health expertise, thereby “contaminating” the control condition. A cluster randomized design, on the other hand, is not feasible since only relatively few CRDs with a non-specialized outpatient clinic for undiagnosed patients exist in Germany.

Secondly, due to the nature of the intervention, participants in the project and staff at the CRDs cannot be blinded to allocated care. However, efforts are taken to divulge medical information only after having obtained informed consent. Nevertheless, a selection bias at enrollment cannot be excluded, but can be tested when comparing the symptoms at baseline as well as the diagnoses established at enrollment with those during the 12-month period thereafter. Furthermore, comparing drop-out rates between the two care models will provide information on potential attrition bias.

The innovative care model combining expertise from both a somatic and a mental health expert in the diagnostic approach of a complex and often persistent unclear symptomatology may not only facilitate and accelerate the process of diagnosis but also help to guide all treatments warranted—may they target a somatic illness, a mental disorder or both. Irrespective of the mental symptoms being the cause of the presenting symptomatology, the consequence of the underlying health condition or unrelated, they can be identified and respective care be initiated. The integration of patients perspectives towards the innovative care model is important to ensure a high level of acceptance for this approach.

## Conclusions

Should the innovative approach in ZSE-DUO prove successful, it will be made available on a wider scale nationally, will be promoted internationally and may serve as a role model for other medical situations.

## Data Availability

Not applicable.
